# Pleural effusion as an isolated finding in COVID-19 infection

**DOI:** 10.1016/j.rmcr.2020.101269

**Published:** 2020-10-21

**Authors:** Mousa Hussein, Irfan Ul Haq, Mansoor Hameed, Merlin Thomas, Anam Elarabi, Mona Allingawi, Issam Al-Bozom

**Affiliations:** Pulmonary Department, Hamad General Hospital, Hamad Medical Corporation, Doha, Qatar

**Keywords:** COVID-19, Pleural effusion, Lactate dehydrogenase, Pleural biopsy

## Abstract

Common radiological findings of COVID -19 infection include bilateral ground-glass opacities in lower lobes with a peripheral distribution. Pleural effusion is considered a rare manifestation of COVID -19 infection. We present a 52 years old patient with a three-week history of right-sided pleuritic chest pain, fever, and dyspnea. Laboratory investigations revealed high *C*-reactive protein and ferritin levels and a positive COVID-polymerase chain reaction (PCR) from a nasopharyngeal swab. Chest X-ray and Computed tomography (CT) identified a moderate right-sided pleural effusion, which was exudative with mixed cellularity and high Lactate dehydrogenase (LDH). Histopathology of thoracoscopic pleural biopsy didn't reveal granulomas, malignancy, or any microbiological growth. We postulate that having ruled out any other cause the effusion was likely related to the Covid-19 infection. Our case highlights that COVID-19 can present with isolated pleural effusions, therefore it should be kept as an etiology of effusions especially if other possible causes have been ruled out.

## 1 Introduction

Coronaviruses are enveloped single-stranded RNA viruses that can affect the lung, liver, and neurological system [1].Its clinical spectrum ranges from asymptomatic to life-threatening acute respiratory distress syndrome. The disease was first identified in Wuhan and has been spreading rapidly worldwide resulting in a global pandemic [2]. A wide variety of clinical manifestations have been reported in patients infected with COVID-19. Common symptoms of Covid-19 include fever, cough, myalgia, fatigue, hemoptysis, headache, nausea, and diarrhea [3]. Chest imaging plays a vital role in diagnosing and assessing the severity and extent of the disease in COVID-19 pneumonia. Most patients with COVID-19 pneumonia have typical imaging features, such as ground-glass opacities alone or in combination with consolidation, vascular enlargement, pneumatocoeles, and traction bronchiectasis [4]. Isolated Pleural disease in the context of COVID-19 is rarely encountered in clinical practice. The common pleural abnormality detected in COVID 19 patients is pleural thickening (15%) followed by pleural effusion (4%) in a study comparing chest CTs from 219 patients with COVID-19 in China and 205 patients with other causes of viral pneumonia in the United States[5].

## 2 Case description

**Fig. 1:**
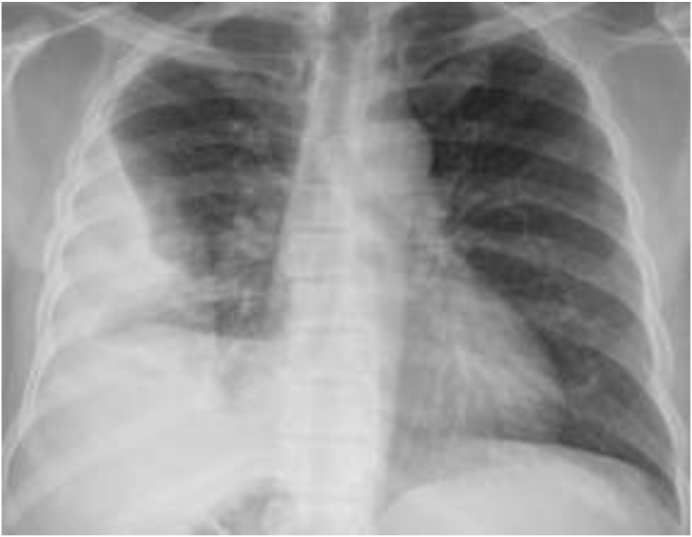
Chest X-ray showing right-sided moderate effusion with thickening.

**Fig. 2:**
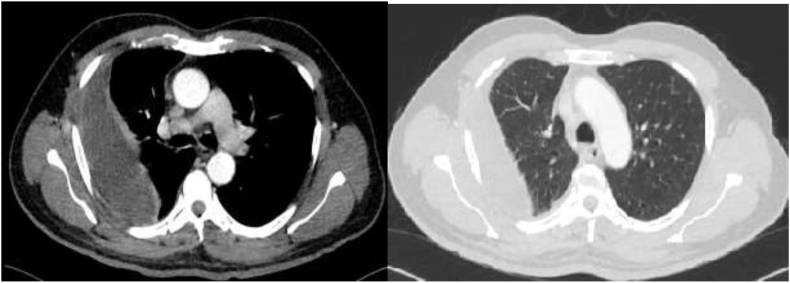
Computed tomography of the chest showing moderate right-sided effusion with normal lung parenchyma.

**Fig. 3:**
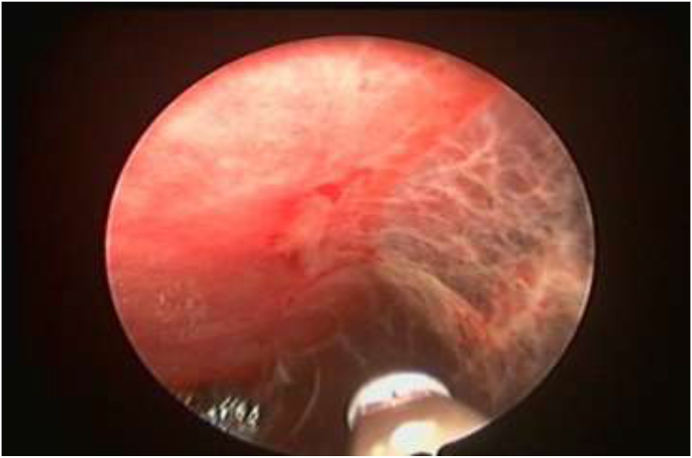
Medical thoracoscopy view, showing inflamed parietal pleura with few adhesions.

**Fig. 4:**
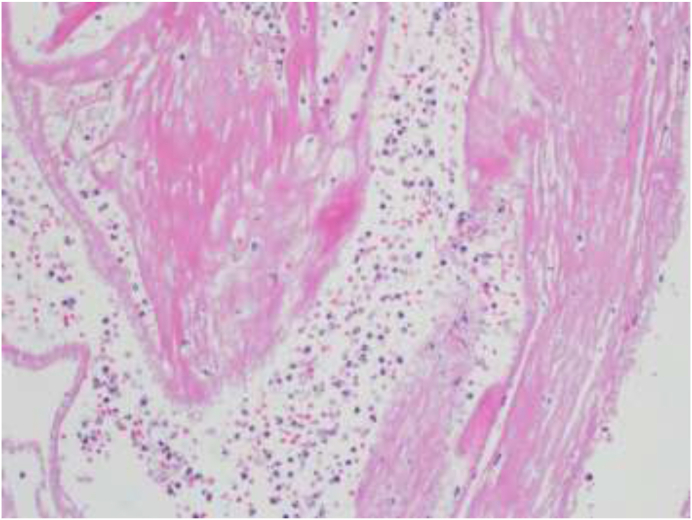
Microscopic view showing acute fibrinous exudate (H&E × 200).

**Fig. 5:**
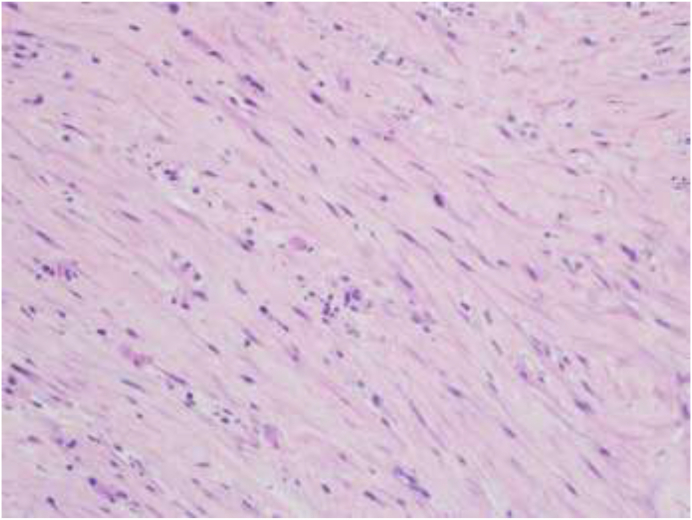
Microscopic view revealing dense fibroblastic reaction characterized by fascicles of spindle cells mixed with fewer numbers of lymphocytes, plasma cells, and eosinophils associated with deposits of dense collagen (H&E × 200).

A 52-year-old male patient, presented with a three weeks history of right-sided chest pain, dyspnea, and fever. The patient denied having night sweats, weight loss, and anorexia. He had no contact with sick patients and no previous history of any respiratory illness. He was non-smoker, with no family history of malignancies. Physical examination revealed decreased breath sounds on the right side. Laboratory results showed a high *C*-reactive protein, ferritin, and liver enzymes along with hyponatremia (Table 1). Chest X-ray and CT scan demonstrated a moderate right-sided pleural effusion, pleural thickening, and normal lung parenchyma (Fig. 1, Fig. 2). A diagnostic pleural tap was performed. The pleural fluid analysis was consistent with an exudative effusion with a pleural fluid PH of 7.5, the glucose of 6.8 mmol/L, and a high LDH of 1185U/L. The pleural fluid differential white cell count showed 45% lymphocytes, 41% neutrophils, and 9% eosinophils. No organism was detected in the pleural fluid including TB on culture and PCR, and the cytology was negative for malignant cells (Table 2). Given the significant community spread of SARS-COV2, a COVID-19 PCR was performed which was positive. Nonetheless, the patient underwent medical thoracoscopy and pleural biopsy to rule out other pathologies as the cause for the effusion. At thoracoscopy visually the parietal pleura was found to be mildly inflamed with a few pleural adhesions (Fig. 3). Histopathological examination showed acute fibrinous exudate consisting of layers of fibrin mixed with abundant neutrophils (Fig. 4). Also seen were areas of dense fibrosis characterized by fascicles of spindle cells mixed with fewer numbers of lymphocytes, plasma cells as well as eosinophils associated with deposits of dense collagen (Fig. 5) with no evidence of granulomas or malignancy. Culture samples of the pleural biopsy were negative for TB. He clinically improved on hydroxychloroquine and antibiotics.

**Table 1:** Relevant lab investigations including infection workup.

Investigation	Result	Normal range
WBC count	11.3	4–10 × 10^3/uL
Platelet count	940	15–400 × 10^3/uL
Hb	11	13-17 gm/dL
Lymphocyte count	2	1–3 x10^3/uL
Creatinine	79	62-106 μmol/L
Sodium	126	136–145 mmol/L
Alanine aminotransferase	71	0–41 U/L
*C*- Reactive protein	240	0–5 mg/L
Procalcitonin	0.17	<0.5 ng/ml
Glucose	5.3	3.3–5.5 mmol/L
Lactate dehydrogenase	214	135–225 U/L
Total protein	76	66-87 gm/L
Ferritin	657	30-533 μg/L
Blood cultures	No growth	–
Urine culture	No growth	–
Common Viruses panel	Negative	–
Sputum AFB smear, PCR, and culture	Negative	–
SARS-Cov 2 PCR	Positive	–
HIV antigen/antibody ELISA	Non-reactive

**Table 2:** Pleural fluid characteristics.

Parameter	Results
Appearance	Turbid
Color	Orange
PH	7.5
Total protein	60 g/dL
Lactate dehydrogenase	1185 U/L
Glucose	6.8 mmol/L
WBC count	2450/mcl (45% Lymphocytes, cells,41% neutrophils and 9% eosinophils)
Microbiology	Negative
Cytology	Few mesothelial cells, numerous inflammatory cells, mainly lymphocytes

## 3 Discussion

COVID-19 has been regarded as a public health emergency of global concern for more than 6 months. With many countries now in the grip of the second wave of COVID-19, unusual presentations of COVID-19 infection are still being reported. Although mortality due to COVID-19 is comparable to MERS and SARS-CoV, its spread and virulence are significantly higher than these viruses [4]. SARS-CoV2 like MERS and SARS-CoV, has a particular affinity for the respiratory tract epithelium.[6] Review of the literature reveals that pleural effusions were seen in one-third of cases with MERS, but were rarelyreported in SARS-CoV [[7], [8]].

Chest imaging is essential for both the initial diagnosis and follow-up. The hallmark radiological findings in COVID-19 patients constitute bilateral peripherally located ground-glass opacities while atypical features include bronchial wall thickening, pleural effusions, and lymphadenopathy [9]. In a systematic review and meta-analysis, the commonest pleural abnormality was pleural thickening (52%) while pleural effusion occurred in only 6% of the cases.10 In our case, the patient developed the pleural effusion and pleural thickening in the absence of any parenchymal lung pathology which is a rare phenomenon. To our knowledge, no relationship has been established between pleural effusions and COVID- 19. However; pleural effusion is rarely encountered in the early phase of COVID-19 infection [10]. Mostly pleural effusions develop after recurrent pneumonia or after the third week of pneumonia [11].

Limited information is available about the characteristic of pleural fluid in COVID-19 patients, due to infection control protocols limiting invasive procedures during the current pandemic. A case series from Singapore describes lymphocytic exudative pleural effusions in three patients with SARS-CoV2. However, one of the patients had a positive pleural fluid culture for Mycobacterium Tuberculosis while the other two were also treated with anti-TB medications as well given a high clinical suspicion and elevated pleural Adenosine deaminase levels[12]. In another recently published study, the pleural fluid characteristics of four patients with COVID-19 pneumonia were reviewed. Two patients had neutrophil predominant exudative effusion while two had lymphocytic exudative effusion with a variable LDH level (284–3,651 U/L) and no evidence of malignancy or tuberculosis.[13] Elevated pleural fluid LDH levels (greater than 1000 IU/L) suggest empyema, malignant effusion, rheumatoid effusion, or pleural paragonimiasis[13,14].

The significantly elevated level of LDH in pleural fluid can be either due to the presence of hemolyzed red blood cells in the pleural fluid or a heightened immune response observed in many SARS-CoV-2 patients with extremely elevated inflammatory markers causing a high cell turnover [15]. In our case although the initial CRP was high other acute phase/Inflammatory reactants like ferritin and LDH were not significantly raised.

Pleural biopsy is often needed when pleural fluid analysis yields inconclusive results to identify the etiology of the pleural effusion. In our case, pleural biopsies were taken given a high index of suspicion of pleural TB. Biopsies have a higher diagnostic yield for TB and other pathologies and the diagnosis are often made, in the correct clinical context [13]. We attributed the pleural effusion in our case to COVID-19 as no other cause for the pleural effusion was identified despite extensive investigations and the patient improved post thoracoscopy and drainage of fluid with no recurrence to date.

## 4 Conclusion

COVID-19 exhibits a diverse range of clinical presentations and our knowledge regarding COVID-19 is constantly evolving. Clinicians need to familiarize themselves with both typical and atypical radiological manifestations of COVID-19. Pleural effusion in COVID-19 infected patients has been reported and may show a high pleural LDH but this finding needs to be confirmed in other studies. Covid-19 should be thought of as a cause of effusions especially in the absence of any other identifiable etiology.

## Declaration of competing interest

The authors declare that they have no known competing financial interests or personal relationships that could have appeared to influence the work reported in this paper.
